# Differential expression of small RNA pathway genes associated with the *Biomphalaria glabrata/Schistosoma mansoni* interaction

**DOI:** 10.1371/journal.pone.0181483

**Published:** 2017-07-18

**Authors:** Fábio Ribeiro Queiroz, Luciana Maria Silva, Wander de Jesus Jeremias, Élio Hideo Babá, Roberta Lima Caldeira, Paulo Marcos Zech Coelho, Matheus de Souza Gomes

**Affiliations:** 1 Grupo de Pesquisa em Biologia do *Schistosoma mansoni* e sua Interação com o Hospedeiro, Centro de Pesquisas René Rachou, Fundação Oswaldo Cruz, Belo Horizonte, Minas Gerais, Brasil; 2 Serviço de Biologia Celular do Departamento de Pesquisas e Desenvolvimento, Fundação Ezequiel Dias, Belo Horizonte, Minas Gerais, Brasil; 3 Grupo de Pesquisa em Helmintologia e Malacologia Médica, Centro de Pesquisas René Rachou, Fundação Oswaldo Cruz, Belo Horizonte, Minas Gerais, Brasil; 4 Laboratório de Bioinformática e Análises Moleculares, Instituto de Genética e Bioquímica, Campus Patos de Minas, Universidade Federal de Uberlândia, Patos de Minas, Minas Gerais, Brasil; Chungnam National University, REPUBLIC OF KOREA

## Abstract

The World Health Organization (WHO) estimates that approximately 240 million people in 78 countries require treatment for schistosomiasis, an endemic disease caused by trematodes of the genus *Schistosoma*. In Brazil, *Schistosoma mansoni* is the only species representative of the genus whose passage through an invertebrate host, snails of the genus *Biomphalaria*, is obligatory before infecting a mammalian host, including humans. The availability of the genome and transcriptome of *B*. *glabrata* makes studying the regulation of gene expression, particularly the regulation of miRNA and piRNA processing pathway genes, possible. This might assist in better understanding the biology of *B*. *glabrata* as well as its relationship to the parasite *S*. *mansoni*. Some aspects of this interaction are still poorly explored, including the participation of non-coding small RNAs, such as miRNAs and piRNAs, with lengths varying from 18 to 30 nucleotides in mature form, which are potent regulators of gene expression. Using bioinformatics tools and quantitative PCR, we characterized and validated the miRNA and piRNA processing pathway genes in *B*. *glabrata*. *In silico* analyses showed that genes involved in miRNA and piRNA pathways were highly conserved in protein domain distribution, catalytic site residue conservation and phylogenetic analysis. Our study showed differential expression of putative Argonaute, Drosha, Piwi, Exportin-5 and Tudor genes at different snail developmental stages and during infection with *S*. *mansoni*, suggesting that the machinery is required for miRNA and piRNA processing in *B*. *glabrata* at all stages. These data suggested that the silencing pathway mediated by miRNAs and piRNAs can interfere in snail biology throughout the life cycle of the snail, thereby influencing the *B*. *glabrata/S*. *mansoni* interaction. Further studies are needed to confirm the participation of the small RNA processing pathway proteins in the parasite/host relationship, mainly the effective participation of small RNAs in regulating their target genes.

## Introduction

Small noncoding RNAs (ncRNAs) are an important class of RNAs that regulate gene expression. Among these, the miRNAs, siRNAs and piRNAs have been considered the most important small RNAs [1, 2]. They are defined by their length of 18 to 30 nucleotides (nt), their biogenesis and their interaction with Argonaute-proteins [3–5]. The miRNAs are conserved regulators of gene expression in animals and plants and participate in countless biological processes, such as cell growth, proliferation and differentiation as well as organismal metabolism and development [6–8]. Mature piRNAs are associated with PIWI proteins in germline cells and mutations in the piRNA pathway genes resulted in germline cells defects in *Caenorhabditis elegans* and mice [9–11].

Post-transcriptional regulation of gene expression by miRNA and piRNAs is normally referred to as transcript silencing, and there are several core and accessory proteins involved. In animals, the miRNA silencing pathway starts in the nucleus with primary microRNA (pri-miRNA) transcripts synthesized from a DNA template by RNA polymerase II [1]. Inside the nucleus the pri-miRNA is cleaved into pre-miRNA by a microprocessor complex mediated by the RNase III Drosha [12, 13]. The pre-miRNA has a hairpin structure of ~ 60 to 120 nucleotides which is exported from nucleus to cytoplasm by Exportin-5 and RAN-GTP [14–16]. In the cytoplasm, the pre-miRNA is cleaved by Dicer, another RNase III, near the terminal loop, producing a double-stranded structure [17, 18]. In animals, Dicer must interact with protein Loquacious to process the pre-miRNA, which can interfere directly in the production of miRNAs [19–21].

The double strand structure processed by Dicer is loaded onto the RNA-induced silencing complex (RISC) by the Argonaute protein [1, 22, 23]. The Argonaute family is composed of two distinct subclades: AGO and PIWI [24]. Members of the AGO clade are associated with miRNAs and siRNAs, whereas PIWI proteins are associated with piRNAs [23, 25]. Argonaute selects the guiding strand of the small RNA to form the RISC complex [26–31]. The RISC complex is composed of other proteins such as Tudor-SN and FMR-1[32].

In the RISC complex, the mature miRNAs regulate the expression of the mRNA targets via 3'UTR binding [33]. When the recognition of mRNAs by the RISC-miRNA complex is perfect, complete mRNA degradation occurs. Normally, this process also occurs in plants. However, the recognition can be non-perfect, in which case only translational repression of the mRNA occurs [34–37].

The biogenesis of piRNAs is not well understood, but studies in animals such as *Drosophila melanogaster*, *C*. *elegans* and mouse have improved the understanding of this mechanism [38]. Silencing mediated by piRNAs is characterized by two major pathways: the primary processing pathway and the ping-pong cycle that amplifies secondary piRNAs [11, 39, 40].

In the primary processing pathway, clusters or transposons containing regions that generate piRNAs are transcribed as antisense single strands, and precursor piRNAs are processed by PIWI proteins; however, the entire process is still incompletely characterized [39]. The ping-pong mechanism most likely involves slicer activity of Aubergine and Argonaute 3, and the transcripts can be sense or antisense [41, 42]. Studies have shown that Piwi proteins act in association with proteins containing a TUDOR domain, and this association is necessary for the normal function of the Piwi protein in the biogenesis of piRNAs [39, 43–45].

Recently, an international consortium sequenced, analyzed and published the whole genome and transcriptome of *B*. *glabrata* [46]. The genome data was deposited in VectorBase (https://www.vectorbase.org/organisms/biomphalaria-glabrata) [46–48]. The study focused on the *B*. *glabrata* genome analysis providing novel details on the biological properties of *B*. *glabrata*. The authors predicted 14,423 gene models. These genes are involved in several biological processes such as stress responses, immune function and regulation of gene expression. Particularly, the authors identified nine genes responsible for miRNAs and piRNAs processing machinery. These pathways may assist in our understanding of the biology of *B*. *glabrata* and its relationship with the parasite *S*. *mansoni*. In Brazil, *B*. *glabrata* is the most important species in the transmission of *S*. *mansoni* [49]. All strains of this species are susceptible to infection by *S*. *mansoni* [50]. Some aspects of this interaction are still poorly explored, including the participation of small RNAs in the biology and interaction between *B*. *glabrata* and the parasite *S*. *mansoni* [50, 51].

Despite the great potential for understanding the parasite life cycle, neither the role of small RNAs in schistosomiasis nor the role of small RNAs in the intermediate host have been well explored. In *S*. *mansoni*, the processing pathway of miRNAs has been characterized with differential gene expression of Argonaute and Dicer genes [52], and putative miRNAs have been identified through cloning, sequencing and bioinformatics techniques; interestingly, the piRNAs machinery was not observed in *S*. *mansoni* [52–54]. Although there are studies in the literature involving gene silencing by siRNAs in *B*. *glabrata* [46, 55, 56], this is the first time that the small RNA processing machinery in *B*. *glabrata* has been characterized and validated.

Our aims were to characterize by bioinformatics techniques and validate by qRT-PCR miRNA/piRNA processing pathway genes in *B*. *glabrata*. We hypothesized that the small RNA processing pathway genes are conserved in *B*. *glabrata* and that the genes participate in *S*. *mansoni* infection by interfering directly with the gene expression profile of the snail.

## Materials and methods

### Conserved domain and phylogenetic analyses of miRNA and piRNA pathway genes

The putative *B*. *glabrata* genes involved in the miRNA and piRNA pathways, their respective proteins sequences and their annotation were retrieved from the *B*. *glabrata* genome database in VectorBase (https://www.vectorbase.org/organisms/biomphalaria-glabrata) (S1 Table). They were underpinned by Pfam (30.0) and CDD databases to search for conserved domains, motifs and active site amino acids. The genes orthologous to Bgl-Argonaute, Bgl-Piwi, Bgl-Dicer and Bgl-Drosha were searched in the NCBI Refseq database using Blastp to obtain their homologs from animal species. The best hits from protostomes and deuterostomes animals and from *S*. *mansoni* host animals were retrieved in amino acid fasta format. Multiple alignments of those sequences were done using ClustalX 2.1 [57], and phylogenetic analyses were carried out using MEGA 5.2 [58]. Phylogenetic trees based on the analyses of those sequences were inferred using the neighbor-joining method [59], and the evolutionary distance was computed using the JTT model [60]. The bootstrap consensus tree inferred from 1000 replicates was used to represent the evolutionary history of the taxon analysed [61].

### Biological samples

*B*. *glabrata* snails (Belo Horizonte strain—056/2012/SECEX/CGEN) were obtained from Moluscario Lobato Paraense at the Rene Rachou Research Center. The snails were chosen according to their sexual stage. Specimens up to 18 days after hatching were considered sexually immature and after 20 days were considered sexually mature. For infection assays, sexually mature snails from 7 to 15 mm in size were infected individually with 10 miracidia of *S*. *mansoni* strain LE and maintained for 3 hours under artificial light. After that, the snails were maintained in an aquarium. A total of 30 snails for each time point, with different *S*. *mansoni* infection time were obtained: 4 hours, 12 hours, 24 hours, 7 days, 15 days, 21 days and 30 days after infection covering all the stages of development of *S*. *mansoni* in the snail. For control, snails were maintained in the same condition without infection for each time point. A group of infected snails was maintained for 38 days, being exposed to artificial light at the end of this period to confirm the cercariae elimination, with an infection success rate of over 74%.

### RNA preparation and quantitative real-time PCR

Total RNA was isolated and real-time RT-PCR assays were performed. Whole snails, including the hemolymph, were frozen in liquid nitrogen, macerated and used for total RNA extraction. After homogenizing with 1 ml Tri Reagent^®^ (Sigma-Aldrich) the samples were treated with TURBO DNA-free (Ambion) and quantified using NanoDrop as recommended by the manufacturer. The RNA quality was analysed with a bioanalyzer (Agilent), and all the samples showed adequate quality. For synthesis of cDNA, we used the High-capacity Kit (Life Technologies^™^) according to manufacturer instructions. The primers used in q-PCR assays are shown in S2 Table, and their products in 2% agarose gels are shown in S1 Fig. All primers were designed using the program GeneRunner^®^. RNA that was not reverse-transcribed and without cDNA was used as a negative control. The qRT-PCR assays were performed using Power SYBR Green Master Mix (Invitrogen). The efficiency for each pair of primers was evaluated using Applied Biosystems 7500 software with serial dilutions of 1:1, 1:2, 1:4, 1:8, 1:16 and 1:32. After efficiency analysis only Bgl-Argonaute, Bgl-Drosha, Bgl-Piwi, Bgl-Exportin-5 and Bgl-Tudor genes presented R^2^ ≥ 0,985 and efficiencies from 90 to 110% following for PCR assays [62]. For all investigated transcripts, three biological replicates were performed and gene expression levels were normalized using the myoglobin transcript according to the 2^-ΔΔCt^ method [63]. Statistical analyses among different groups for each gene were performed using the One-Way Anova, with Tukey test as post-hoc test on GraphPad Prism^®^ 5.0 (GraphPad Software, Inc., San Diego, USA). Values of p ≤ 0.05 were considered statistically significant and are denoted with asterisks in the figures.

### Sanger sequencing

Sanger sequencing of PCR products was performed for primers validation [64, 65]. The CAP3 algorithm (http://doua.prabi.fr/software/cap3) was used for sequence assembly and contig analysis [66]. Blastn and Clustal X 2.1 were used to analyze and confirm the miRNAs and piRNA pathway genes amplification.

## Results

### Characterization of putative miRNAs and piRNA pathway proteins: Alignment and phylogenetic analysis

The putative *B*. *glabrata* proteins involved in the miRNAs and piRNA pathways were retrieved from Vectorbase and used to find homologous proteins from other organisms in the NCBI RefSeq protein database. We selected Bgl-Argonaute (S3 Table), Bgl-Piwi (S4 Table), Bgl-Drosha (S5 Table) and Bgl-Dicer (S6 Table) for further *in silico* analysis. The best orthologous protein Blast hits from Protostomes, Deuterostomes and *S*. *mansoni* host species were retrieved for further analyses.

**Fig 1:**
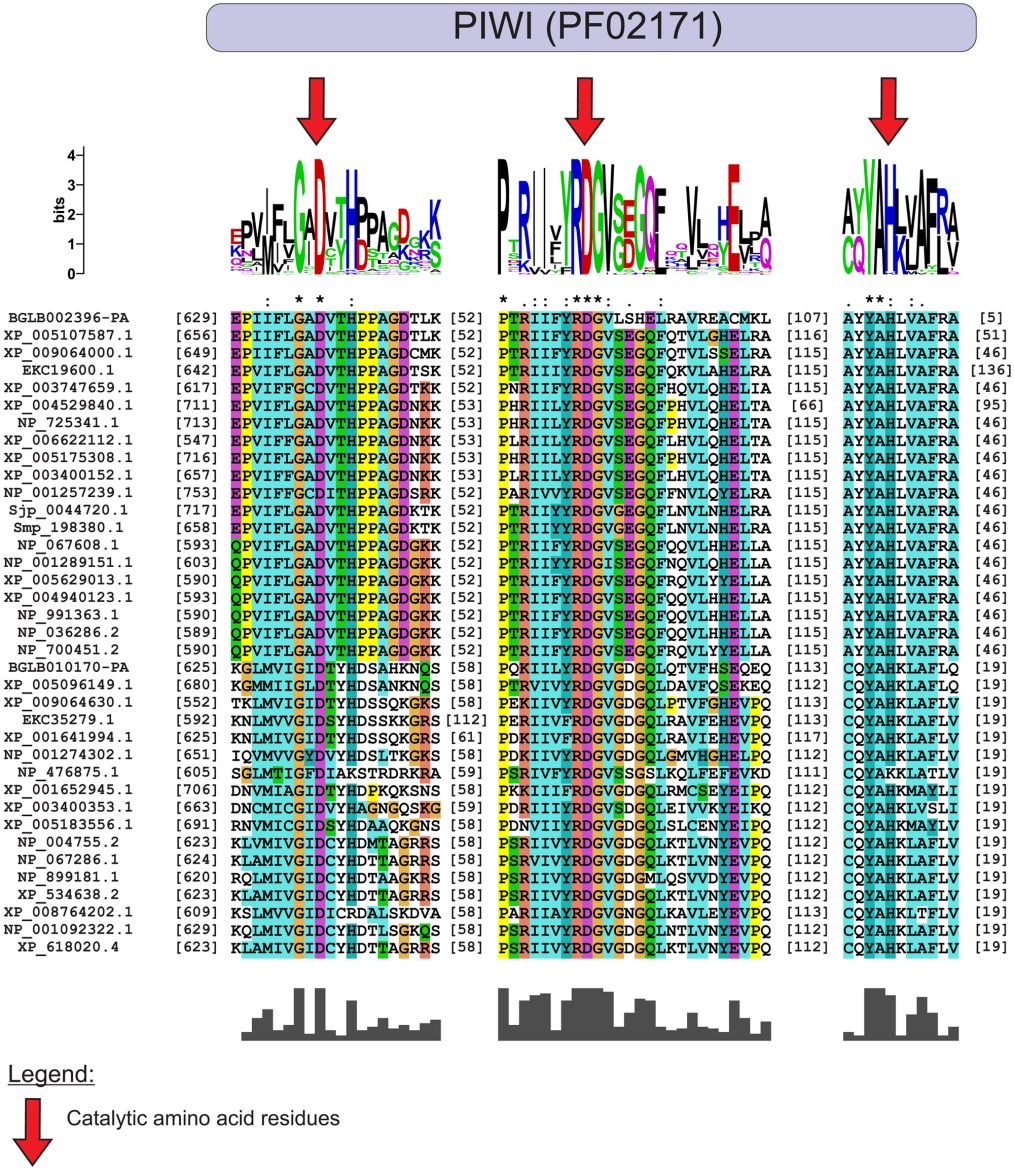
Multiple alignment of the PIWI domain of Ago-like and Piwi-like proteins. The highlighted residues, D (aspartic acid) and H (Histidine), which are highly conserved in the PIWI domains of *B*. *glabrata* and their orthologues, are responsible for the catalytic activity. The sequences used for the Ago-like family were as follows: BGLB002396-PA (*Biomphalaria glabrata*), XP_005107587.1 (*Aplysia californica*), XP_009064000.1 (*Lottia gigantea*), EKC19600.1 (*Crassostrea gigas*), XP_003747659.1 (*Metaseiulus occidentalis*), XP_004529840.1 (*Ceratitis capitata*), NP_725341.1 (*Drosophila melanogaster*), XP_006622112.1 (*Apis dorsata*), XP_005175308.1 (*Musca domestica*), XP_003400152.1 (*Bombus terrestris*), NP_001257239.1 (*Caenorhabditis elegans*), Sjp_0044720.1 (*Schistosoma japonicum*), Smp_198380.1 (*Schistosoma mansoni*), NP_067608.1 (*Rattus norvegicus*), NP_001289151.1 (*Danio rerio*), XP_005629013.1 (*Canis lupus familiaris*), XP_004940123.1 (*Gallus gallus*), NP_991363.1 (*Bos taurus*), NP_036286.2 (*Homo sapiens*) and NP_700451.2 (*Mus musculus*). The sequences used for the Piwi-like family were as follows: BGLB010170-PA (*Biomphalaria glabrata*), XP_005096149.1 (*Aplysia californica*), XP_009064630.1 (*Lottia gigantea*), EKC35279.1 (*Crassostrea gigas*), XP_001641994.1 (*Nematostella vectensis*), NP_001274302.1 (*Hydra vulgaris*), NP_476875.1 (*Drosophila melanogaster*), XP_001652945.1 (*Aedes aegypti*), XP_003400353.1 (*Bombus terrestris*), XP_005183556.1 (*Musca domestica*), NP_004755.2 (*Homo sapiens*), NP_067286.1 (*Mus musculus*), NP_899181.1 (*Danio rerio*), XP_534638.2 (*Canis lupus familiaris*), XP_008764202.1 (*Rattus norvegicus*), NP_001092322.1 (*Gallus gallus*) and XP_618020.4 (*Bos taurus*).

**Fig 2:**
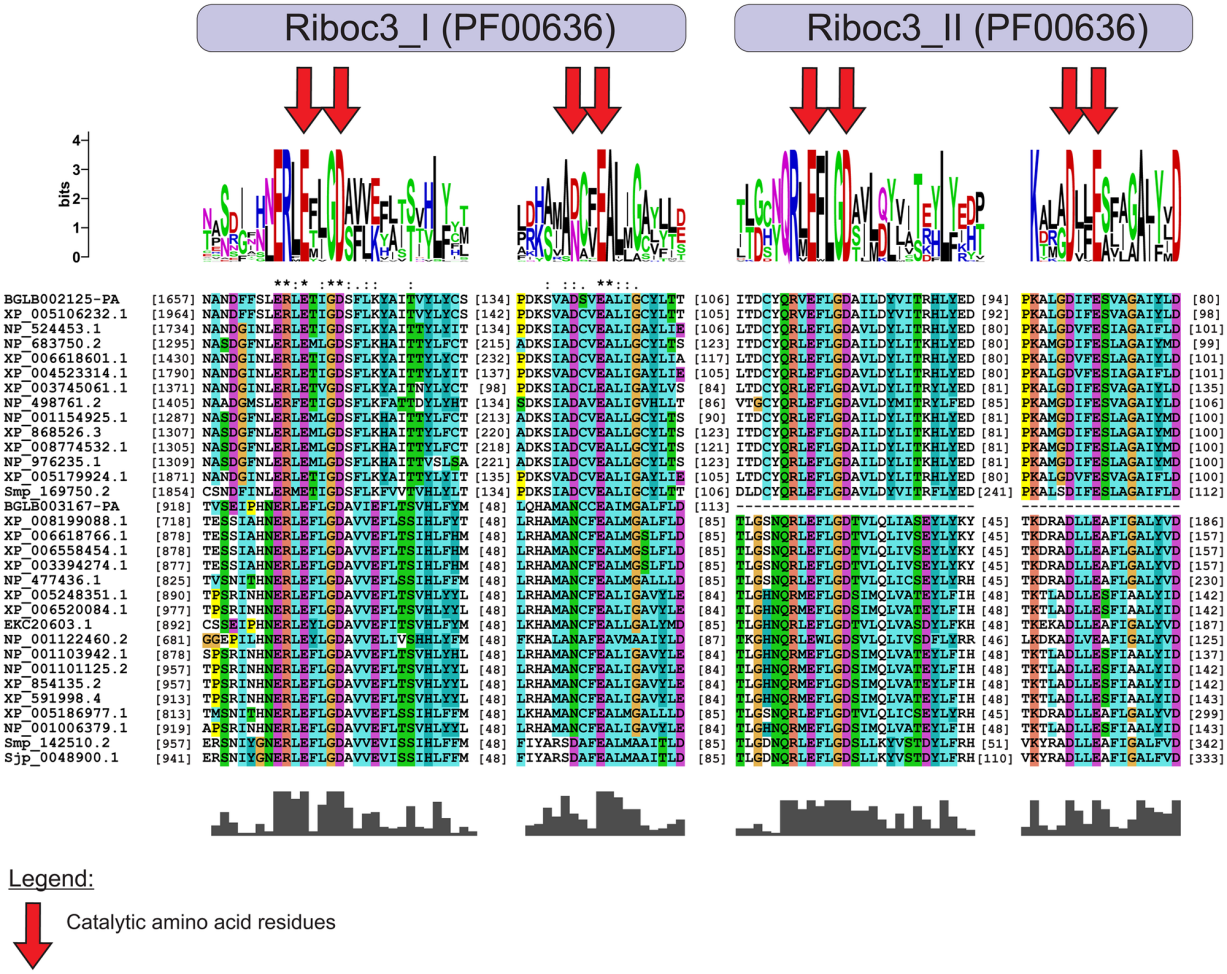
Multiple alignment of the RIBOc domain of Drosha and Dicer proteins. The highlighted residues, D (aspartic acid) and E (glutamic acid), which are highly conserved in the RIBOc domain of *B*. *glabrata*, and their orthologues are responsible for its catalytic activity. The sequences used for the Dicer family were as follows: BGLB002125-PA (*Biomphalaria glabrata*), XP_005106232.1 (*Aplysia californica*), NP_524453.1 (*Drosophila melanogaster*), NP_683750.2 (*Mus musculus*), XP_006618601.1 (*Apis dorsata*), XP_004523314.1 (*Ceratitis capitata*), XP_003745061.1 (*Metaseiulus occidentalis*), NP_498761.2 (*Caenorhabditis elegans*), NP_001154925.1 (*Danio rerio*), XP_868526.3 (*Canis lupus familiaris*), XP_008774532.1 (*Rattus norvegicus*), NP_976235.1 (*Bos taurus*), XP_005179924.1 (*Musca domestica*) and Smp_169750.2 (*Schistosoma mansoni*). The sequences used for the Drosha family were as follows: BGLB003167-PA (*Biomphalaria glabrata*), XP_008199088.1 (*Tribolium castaneum*), XP_006618766.1 (*Apis dorsata*), XP_006558454.1 (*Apis mellifera*), XP_003394274.1 (*Bombus terrestris*), NP_477436.1 (*Drosophila melanogaster*), XP_005248351.1 (*Homo sapiens*), XP_006520084.1 (*Mus musculus*), EKC20603.1 (*Crassostrea gigas*), NP_001122460.2 (*Caenorhabditis elegans*), NP_001103942.1 (*Danio rerio*), NP_001101125.2 (*Rattus norvegicus*), XP_854135.2 (*Canis lupus familiaris*), XP_591998.4 (*Bos taurus*), XP_005186977.1 (*Musca domestica*), NP_001006379.1 (*Gallus gallus*), Smp_142510.2 (*Schistosoma mansoni*) and Sjp_0048900.1 (*Schistosoma japonicum*).

Our studies showed that the *B*. *glabrata* small RNA pathway proteins Bgl-Argonaute and Bgl-Piwi have the same catalytic site amino acid residues involved in slicer function as the model organisms, *D*. *melanogaster* and *C*. *elegans*. In the alignments of Bgl-Argonaute and Bgl-Piwi (Fig 1) and their orthologous proteins, the catalytic amino acids DDH (aspartic acid, aspartic acid and histidine) of the PIWI domain, which are important for cleavage of longer miRNA precursors [67, 68], were highly conserved. Bgl-Drosha and Bgl-Dicer (Fig 2) had RIBOc domains with conserved residues EDDE (glutamic acid, aspartic acid, aspartic acid, glutamic acid), which are important for the cleavage of miRNA precursors [13, 69]. The sequence for Bgl-Drosha did not contain a second RIBOc domain, probably due to lack of coverage in the sequencing of this genome region. Future genome versions might solve this issue. These results provide strong evidence that small RNAs pathway genes are present in the *B*. *glabrata* genome and play a similar function in the snail cells compared to their orthologues.

**Fig 3:**
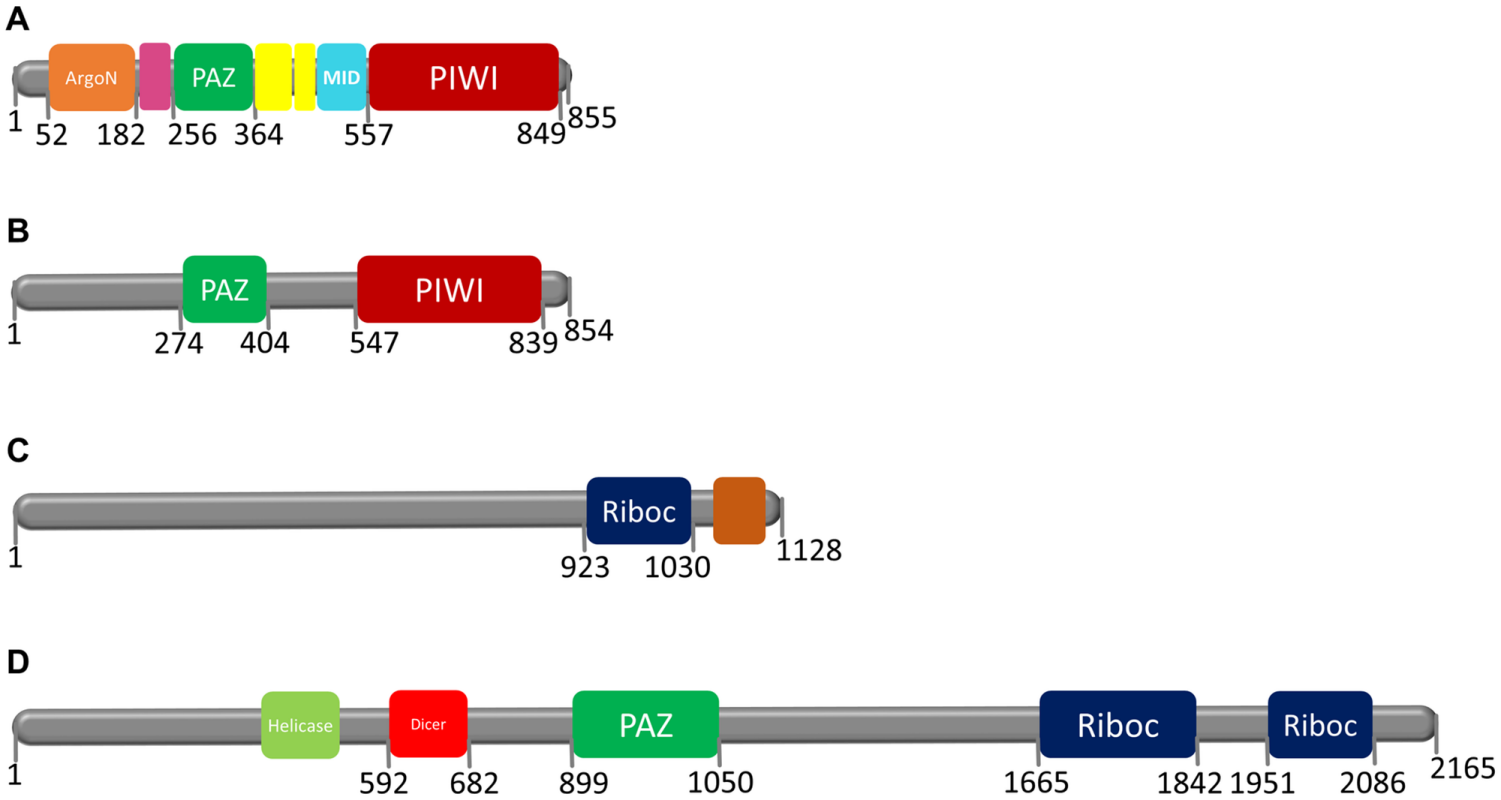
Domain structure of small RNA processing pathway proteins in *B*. *glabrata*. **A—Bgl-Argonaute** has the following domains: ArgoN (PF16486—position 52 to 182 and E-value 8.5e^-31^), ArgoL1 (PF08699—position 192 to 242 and E-value 9.8e^-24^), PAZ (PF02170—position 256 to 364 and E-value 8.7e^-18^), ArgoL2 (PF16488—position 373 to 419 and E-value 1.6e^-12^), ArgoL2 (PF16488—position 424 to 460 and E-value 4.4e^-12^), ArgoMid (PF16487—position 470 to 550 and E-value 2.4e^-34^) and PIWI (PF02171—position 557 to 849 and E-value 4.7e^-104^); **B—Bgl-Piwi** has the following domains: PAZ (PF02170 –position 274 to 404 and E-value 1.3e^-32^) and PIWI (PF02171—position 547 to 839 and E-value 6.2e^-98^); **C—Bgl-Drosha** has the following domains: Ribonucleas_3_3 (PF14622—position 923 to 1030 and E-value 1.3e^-21^) and DSRM (PF00035—position 1081 to 1124 and E-value 2.00e^-06^); **D—Bgl-Dicer** has the following domains: Helicase_C (PF00271—position 423 to 503 and E-value 5.4e^-12^), Dicer_dimer (PF03368—position592 to 682 and E-value 1.3e^-24^), PAZ (PF02170—position899 to 1050 and E-value 1.2e^-33^), Ribonuclease_3 (PF00636 –position 1665 to 1842 and E-value 8.2e^-34^) and Ribonuclease_3 (PF00636—position1951 to 2086 and E-value 9.7e^-22^).

The proteins Bgl-Argonaute (855 aa), Bgl-Piwi (854 aa), Bgl-Drosha (1128 aa) and Bgl-Dicer (2165 aa) were analysed using the Pfam database, and they displayed a conserved domain distribution compared to their orthologues (Fig 3).

**Fig 4:**
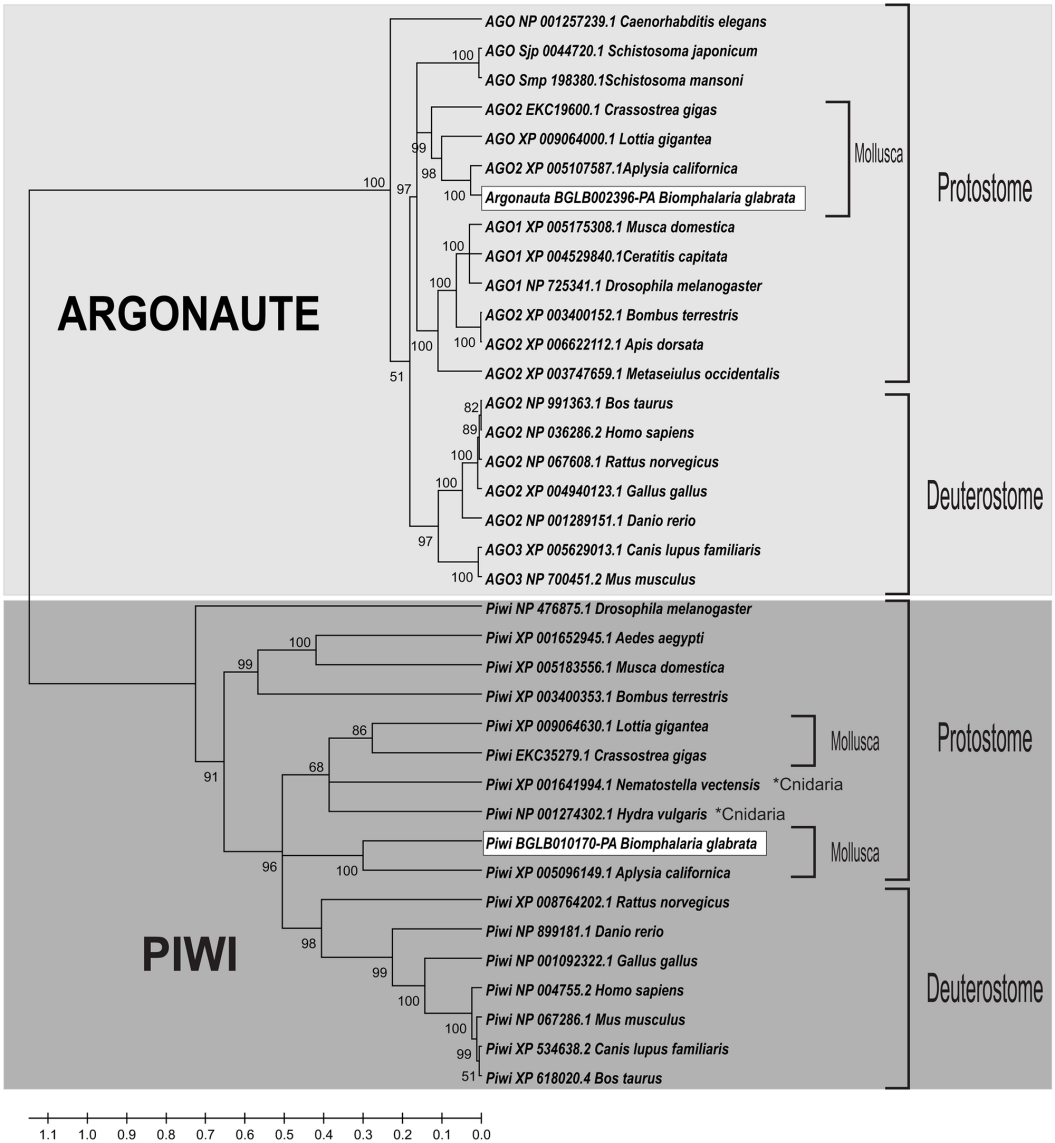
Phylogenetic tree of Bgl-Argonaute and Bgl-Piwi with their orthologues. The phylogenetic analysis was performed using Mega 5.2 with bootstrap analysis. Bootstrap percentages are indicated at each branch.

**Fig 5:**
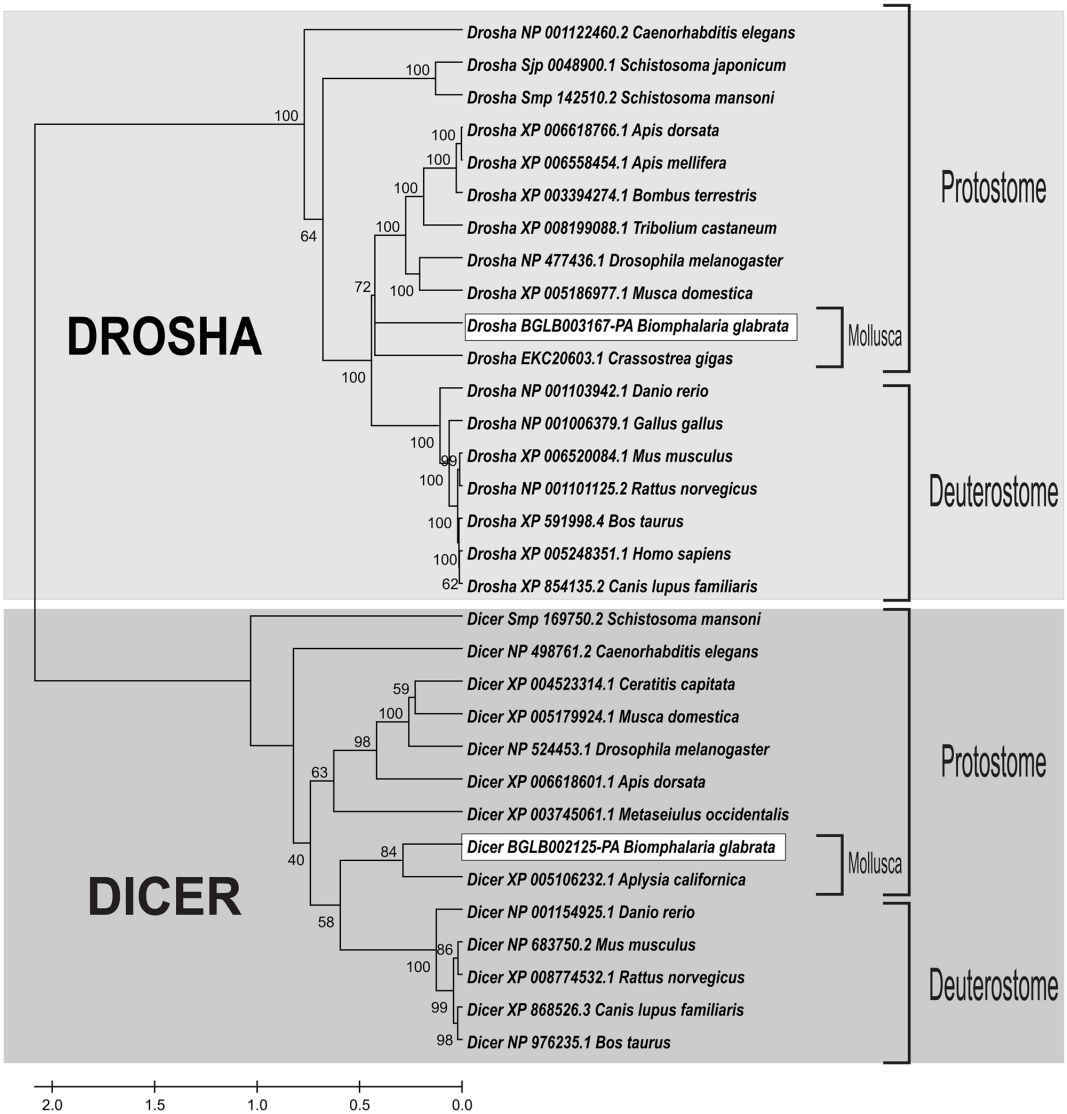
Phylogenetic tree of Bgl-Drosha and Bgl-Dicer with their orthologues. The phylogenetic analysis was performed using Mega 5.2 with bootstrap analysis. Bootstrap percentages are indicated at each branch.

To evaluate the evolutionary history of *B*. *glabrata* small RNA pathway proteins, a phylogenetic tree was generated using the neighbor-joining method with high bootstrap values (1000). Bgl-Argonaute, Bgl-Piwi, Bgl-Dicer and Bgl-Drosha clustered with their respective orthologous proteins, indicating strong structural conservation of these proteins. The amino acid sequences demonstrated evident separation between clades in both Argonaute family proteins, Argonaute-like and Piwi-like proteins, and RNAse III family proteins, Dicer-like and Drosha-like proteins. As shown in Fig 4, Bgl-Argonaute and Bgl-Piwi clustered with their respective orthologous proteins from *Aplysia californica*, *Lottia gigantea* and *Crassostrea gigas* species forming a Mollusca clade. The RNase III Drosha and Dicer proteins (Fig 5) clustered with their orthologues from Mollusca species. Bgl-Drosha formed a clade with *C*. *gigas* and Bgl-Dicer with *A*. *californica*. These results confirmed that these putative *B*. *glabrata* proteins are, in fact, true orthologues and likely maintain their function in the various species.

### Relative gene expression profile analysis by quantitative real-time PCR

**Fig 6:**
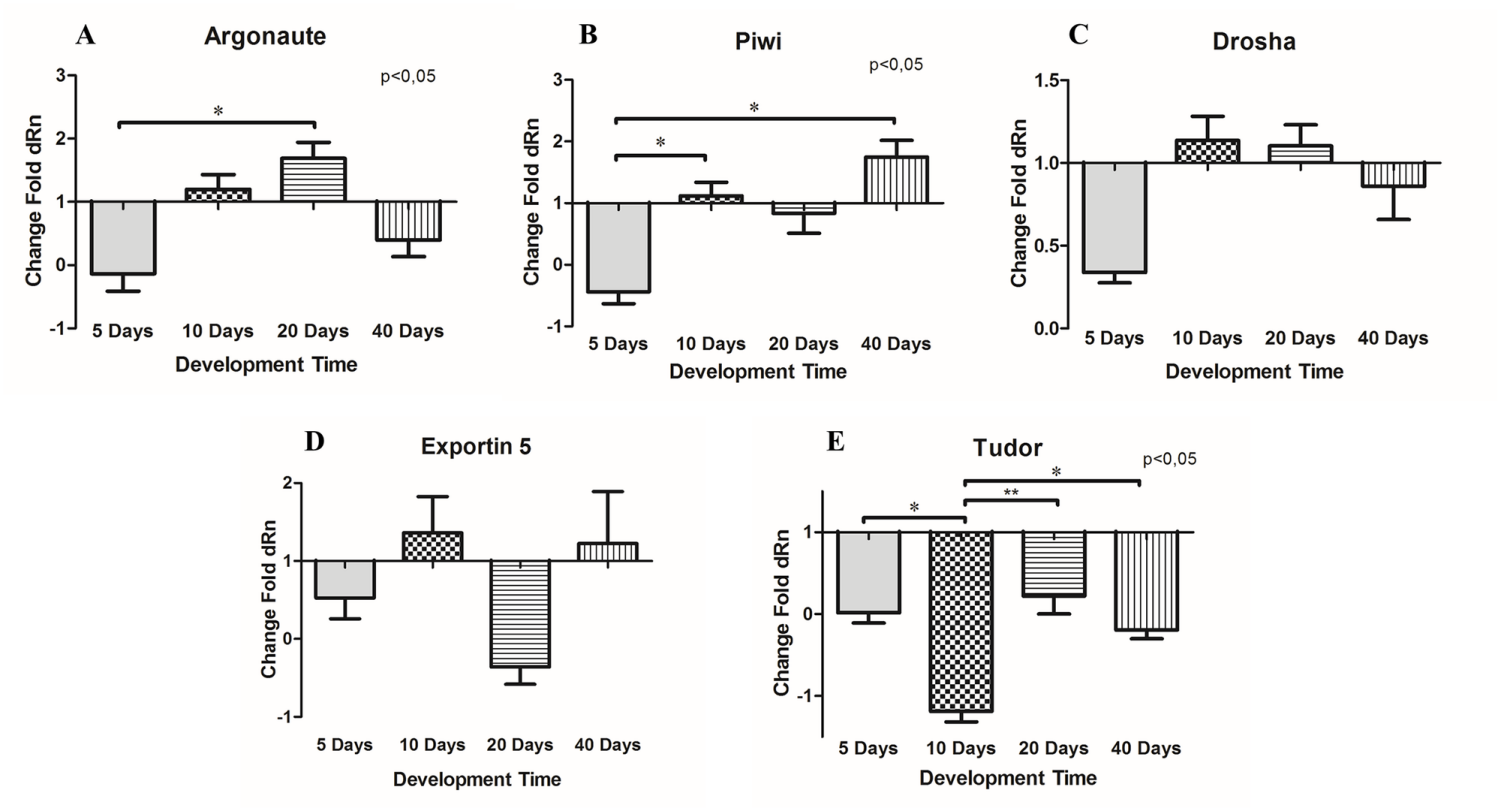
Relative gene expression for several development times in *B*. *glabrata* using the time of mass eggs as the baseline of assay for all genes. **A—Argonaute** was significantly up-regulated at 20 days of development compared to 5 days; **B—Piwi** was significantly up-regulated at 10 and 40 days compared to 5 days; **C—Drosha** levels were not significantly different among the groups; **D—Exportin-5** was not significantly different among the groups; **E—Tudor** was significantly down-regulated at 10 days compared to all other times.

After characterizing *B*. *glabrata* small RNAs genes by bioinformatics analyses, we validated the results using real-time PCR targeting five genes, Bgl-Argonaute, Bgl-Piwi, Bgl-Drosha, Bgl-Exportin-5 and Bgl-Tudor. Initially we aimed to uncover the gene expression profiles of these genes across several stages of snail development. For that, we selected the following development times: egg mass, and 5, 10, 20 and 40 days after hatching. The first time point was used as a control. These times were selected to include sexually immature and mature snails to cover a wide span of the life cycle. The sexual maturation of *B*. *glabrata* is very variable, but in general, the snail is considered mature when oviposition begins, which normally starts at 7 weeks of life, as was reported by Pimentel (1957) [70]. In the Moluscario Lobato Paraense, we observed that *B*. *glabrata* displays sexual maturation after 20 days, which is different of other species, such as *B*. *tenagophila*, which displays sexual maturation from 40 to 47 days. Based on these observations, time points at 5 and 10 days were chosen to represent immature snails, and the 20 day time point to represent the beginning of sexual maturation. These times represent an important range of snail development and, all these were compared to egg mass as control. Thus, we were able to confirm differential gene expression at different *B*. *glabrata* development times. The relative expression of genes (Fig 6) displayed an interesting profile that showed the important participation of these genes in snail development. The Bgl-Tudor profile was very intriguing because this gene was always down regulated. As Tudor is an important gene in both miRNA and piRNA pathways [43, 44] its role is probably strongly linked to regulating the gene expression of small RNA machinery genes in *B*. *glabrata*.

**Fig 7:**
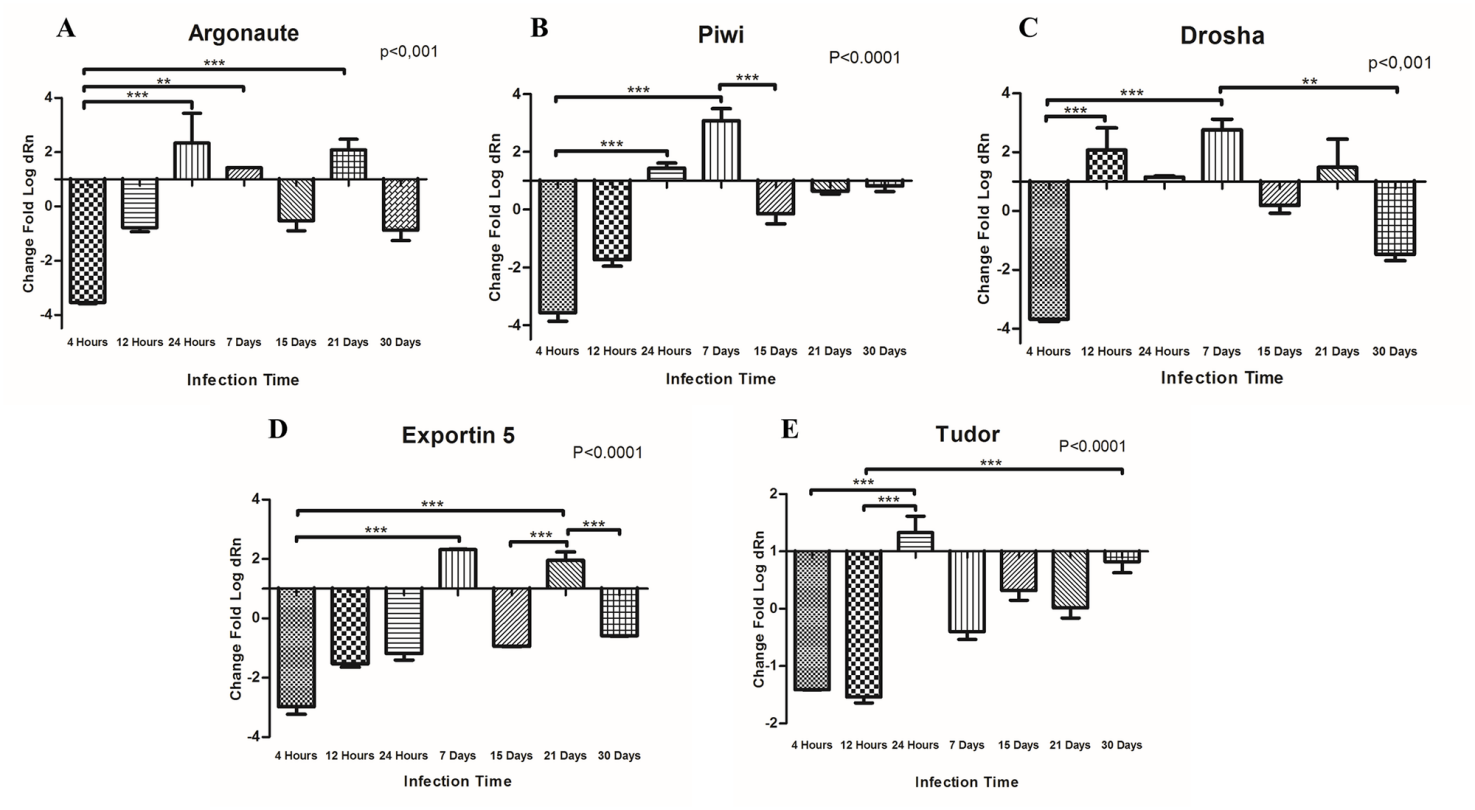
Relative gene expression of *B*. *glabrata* at several time points of infection by *S*. *mansoni* using uninfected snails at the same time points as a baseline. **A—Argonaute** was significantly down-regulated 4 hours after infection compared to 24 hours, 7 days and 21 days after infection; **B—Piwi** was significantly down-regulated at 4 hours compared to 24 hours and 7 days but was significantly up-regulated at 7 days compared to 15 days; **C—Drosha** was significantly down-regulated at 4 hours compared to 12 hours and 7 days but was significantly up-regulated at 7 days compared to 30 days; **D—Exportin-5** was significantly down-regulated at 4 hours compared to 7 days and 21 days; and significantly up-regulated at 21 days compared to 15 and 30 days; **E—Tudor** was significantly down-regulated at 4 hours compared to 24 hours and significantly down-regulated at 12 hours compared to 24 hours and 30 days.

After confirming the importance of the small RNA pathway in snail development, we asked the following: does the infection by *S*. *mansoni* interfere with the gene expression of small RNA pathway genes in *B*. *glabrata*? For this, we proposed to study seven chronologically important moments during the process of infection by *S*. *mansoni*. The first moment was 4 hours after infection because it marks the first reactions of snail to *S*. *mansoni* penetration. The next 12 and 24 hours are important because they represent the first *S*. *mansoni* transformation within the snail body. At 7 days, *S*. *mansoni* uses several strategies to escape the immunologic responses of the snail [71, 72]. At 15 and 21 days, which marks a remarkable period for the interaction, the parasites undergo modifications to transition from primary to secondary sporocysts and start moving to the digestive gland of the snail. At 30 days, the snail begins the cercariae elimination. All these time points are well known and were chosen to cover a wide range of development stages of the parasite within the snail host [71–74]. Duplicate control groups for all infection time points were included using uninfected snails to control for gene expression. We observed differential expression for Bgl-Argonaute, Bgl-Drosha, Bgl-Piwi, Bgl-Exportin-5 and Bgl-Tudor throughout all infection times (Fig 7). The behaviour of *B*. *glabrata* genes at different time points during infection was very interesting, especially at 4 hours after infection, when all the genes showed strong down-regulation. The migration and transformation of parasites within snails conferred similar expression profiles among genes.

### Sequencing of the PCR products

The PCR products were sequenced by the Sanger method. This was important to confirm the amplicon used to measure gene expression. For analysis, we used the CAP3 algorithm (http://doua.prabi.fr/software/cap3). The contigs (S7 Table) were compared using the Blastn and RefSeq NCBI databases, using only the reference organisms *D*. *melanogaster* and *C*. *elegans*. All genes were the same as designed, confirming the *B*. *glabrata* genome sequence.

## Discussion

Recently, Adema et. al (2017), in the whole *B*. *glabrata* genome paper, predicted the putative small RNA pathway genes in the international collaborative work consortium among researches worldwide, aiming to decode the snail genome. In our study, instead of prediction through sequencing and *in silico* analysis, we had characterized and validated those genes for putative small RNA pathway in different snail life times and in infection conditions through RT-qPCR study. Additionally, we identified and validated another important gene involved in miRNA biogenesis, the Exportin-5, which was not identified in genome initiative.

In *B*. *glabrata*, we showed the participation of small RNAs pathway genes at crucial moments of the snail life cycle and in interaction with the *S*. *mansoni* parasite. First, we retrieved the *B*. *glabrata* small RNA pathway genes and their respective putative proteins from VectorBase. The Bgl proteins were compared with their orthologues from different organisms, showing high similarity at the amino acid level. We showed that Bgl-Argonaute and Bgl-Piwi had 855 and 854 amino acids, respectively, and the PIWI domain had, in both, 292 amino acids with catalytic residues DDH [1, 67]. These residues are highly conserved and very important for slicer activity of the PIWI domain [68, 75, 76]. They are responsible for cleaving the complementary strand, preserving the strand guide and binding the magnesium ion [77, 78]. The concentration of this ion is critical for the function of the silencing process [77].

Proper function of the small RNA processing pathway depends directly on the RNases III enzymes, Drosha and Dicer and their absence might promote the occurrence of numerous disorders [69, 79]. In *B*. *glabrata*, the sequence of the putative Drosha gene might not be complete because a second Riboc domain was not found. Bgl-Drosha had a sequence of only 1128 amino acids. Other sequencing attempts may be necessary to complete this missing region. However, the first Riboc domain contained all the important catalytic residues D and E (aspartic acid and glutamic acid) [12, 13, 80]. Drosha, in association with DGCR8, has been identified as important for cleavage of pri-miRNA to pre-miRNA [13]. We were not able to identify and characterize DGCR-8 gene in *B*. *glabrata*.

The alignment and phylogenetic tree of Bgl-Dicer confirmed the same RNase III properties found in their orthologues. Bgl-Dicer has 2165 amino acids and two complete Riboc domains. The important residues, EEDD in the first domain and QEDDE in the second domain, demonstrated the capacity of Bgl-Dicer to bind with magnesium ions and cleave the pre-miRNAs to create a double strand RNA, miRNA duplex [13, 69, 79].

The results confirmed that *B*. *glabrata* has the genes involved in miRNA and piRNA processing pathways and that these genes have all the domains and amino acids residues important for normal function as observed in their orthologous organisms. To validate these genes, we performed real-time PCR assays and sequencing. We determined the gene expression profile of some miRNA and piRNA pathway genes at different time points in the snail life cycle. We also evaluated the role of infection by *S*. *mansoni* on the expression of *B*. *glabrata* miRNA and piRNA pathway genes. The studies involving small RNA pathway genes were previously performed in the *S*. *mansoni* and some genes displayed differential expression during schistosomula development [52, 53], but, for that time none of the genes was characterized in the vector.

As showed in S1 Fig, all the tested genes were expressed in *B*. *glabrata*. However, only Bgl-Argonaute, Bgl-Piwi, Bgl-Drosha, Bgl-Tudor and Bgl-Exportin-5 genes were tested because they were the only ones to meet the requirements for real-time PCR, including amplification efficiency [62]. The gene expression profile at different time points in the snail life cycle (5, 10, 20 and 40 days of snail development time) showed strong variation among the samples, suggesting important roles for the small RNA pathway genes at several points in the life cycle of *B*. *glabrata*.

Bgl-Argonaute was significantly up-regulated at 20 days of development compared with 5 days (Fig 6A). This indicates that a larger demand for miRNAs occurs during the sexual maturation process. A similar profile was observed for Bgl-Drosha (Fig 6C), but at the same time, Bgl-Exportin-5 (Fig 6D) was strong down-regulated. Exportin-5 is responsible for exporting the pre-miRNA from nucleus to the cytoplasm [14, 15] and up-regulation of Bgl-Drosha and Bgl-Argonaute was expected to correlate with Bgl-Exportin-5 up-regulation. Taken together, these data suggests that in *B*. *glabrata* the expression of miRNAs processing pathway genes probably is not synchronized. Bgl-Argonaute and Bgl-Drosha (Fig 6A and 6C) were up-regulated at 10 and 20 days, showing relation to snail sexual maturation, since oviposition begins at 20 days. The role of Argonaute in gene silencing has been already shown as important for animals development [68, 81, 82] and, Drosha activity is essential for the animal survival [83], but only Argonaute gene was significantly up-regulated at day 20 compared to day 5.

Previous findings has already evidenced that PIWI protein is associated with reproduction and is mainly presents in germ line cells, acting in the control of transposons [41, 84–88]. Our results are consistent with those findings, since we showed that Bgl-Piwi was up-regulated at 40 days of development (Fig 6B), the period of the highest egg production in the snails studied. In contrast, Tudor, which is closely related to Piwi and involved in the primary processing of piRNAs and repression of transposons [44, 89–92], was down-regulated in *B*. *glabrata* at all times analysed, suggesting the intriguing possibility that this gene might participate in the piRNA pathway of *B*. *glabrata*. However, Tudor is related to the control of gene transcription by epigenetic processes [44, 89]. Perhaps the consistent down-regulated profile is related to the need to maintain the expression of important genes for snail development, or it may be related to the regulation of both miRNA and piRNA pathways [32].

Bgl-Piwi (Fig 6B) was significantly down-regulated at 5 days compared to 10 and 40 days, showing the importance of this gene in adult stages of snails. This time point has special importance, because there is a large production of eggs, and Piwi is strongly associated with reproductive periods in animals [86]. Bgl-Exportin-5 (Fig 6D) was up-regulated at 10 and 40 days, representing juvenile and adult stages respectively and, thus reinforcing the idea that miRNAs are very important at all the stages of development, considering that Exportin-5 is crucial for their production [32]. At all times, Bgl-Tudor showed the same profile, except at 10 days, when it was strongly down-regulated. Tudor is important for processing miRNAs [44] and mainly piRNAs, and this expression pattern reflects the important role of Bgl-Tudor in the snail.

After confirming that the small RNA pathway is important for snail development, we also evaluated the influence of infection of *B*. *glabrata* by *S*. *mansoni* on gene expression of miRNA and piRNA pathway genes. Regarding to this, we observed that at 4 hours after infection, all the genes showed a resilient subexpression (Fig 7). At this specific time point, the miracidia, ciliated larval stage of *S*. *mansoni*, have already transitioned into primary sporocysts, still retained near the penetration site [72, 73] and their presence may interfere with the gene expression of small RNAs machinery. It was already known that the moment of parasite penetration in the snail is marked by considerable damage to the snail tissues and also by intense morphological and functional modifications of the parasite to survive in the hostile environment [74], regulation of small RNA pathway genes is probably one of the main mechanisms orchestrating this relationship.

Our results showed a highly variable profile of expression between 12 hours and 7 days after infection for all genes. These findings reinforced the idea that the establishment of infection is highly traumatic to the snail, and it can even lead it to death [71, 72]. In this period, *S*. *mansoni* develops several strategies to scape the immune system of the snail, including mimicking membrane molecules of the snail [93, 94]. The actions of the parasite probably greatly influence the expression of the machinery of small RNA genes, favoring the infection process.

A new, important event was observed at 15 and 21 days after infection for all the genes, which is probably due to the immune system of the snail responding to *S*. *mansoni* transitioning from primary sporocysts to secondary sporocysts [73, 74, 95]. During this period, intense transformation and migration of sporocysts to the digestive gland occurs [72, 95], and miRNA machinery genes were down-regulated at 15 days and up-regulated at 21 days. On the other hand, the piRNA machinery genes Bgl-Piwi and Bgl-Tudor were down-regulated, suggesting that the under-expression of piRNAs favors the susceptibility of *B*. *glabrata* by *S*. *mansoni*.

Another important point was the fact that the Bgl-Argonaute, Bgl-Drosha and Bgl-Exportin-5 genes, which are associated with miRNA machinery, showed strong subexpression at 30 days after infection, whereas Bgl-Piwi and Bgl-Tudor genes, which are associated with the piRNA machinery, had only slightly down-regulated profile. Previous studies have shown the absence of piRNAs in *S*. *mansoni* [52, 53]. Therefore, it is possible that there is a relationship between the susceptibility of *B*. *glabrata* to infection by *S*. *mansoni* and the piRNAs machinery.

These results strongly suggested important roles for small RNAs in the *S*. *mansoni* infection process, directly affecting the relationship susceptibility/resistance of *B*. *glabrata* to *S*. *mansoni*. The role of miRNAs in different infection processes has been widely studied and showed to promote or inhibit the infection [96–98]. Our results suggested that genes of the miRNA and piRNA machinery, when down-regulated, favors susceptibility of *B*. *glabrata* to infection by *S*. *mansoni*, and probably some small RNAs are closely involved with susceptibility/resistance during this process. However, more additional studies need to be made to understand better this relationship.

## Conclusion

Our data showed that the silencing pathways mediated by miRNAs and piRNAs are present in *B*. *glabrata* and, is able to interfere in snail biology throughout its life cycle, by interfering in the *B*. *glabrata/S*. *mansoni* interaction process. Complementary studies are already being conducted by our research group aiming to confirm the participation of the predicted proteins of the miRNA and piRNA pathway in the parasite/host relationship. Our particular interest on this subject is reinforced by the fact of *S*. *mansoni* does not express piRNAs, and further the genes involved in the piRNAs biogenesis are present in *B*. *glabrata*, but they show significantly down-regulation at crucial moments of infection, such as wile worm transformation to sporocysts stage as well as its migration through the snail body of sporocysts.

## Supporting information

S1 Fig Agarose gel 2% for evaluation the PCR products of primers the machinery the miRNAs and piRNAs in *B*. *glabrata*. **A**: 1- Molecular weight marker, 2- Argonaute, 3- Dicer, 4- Drosha, 5- FMR, 6- Loquacious, 7- TDRD, 8- molecular weight marker. **B**: 1- Molecular weight marker, 2- Piwi, 3- Tudor, 4- SPN, 5- Exportin-5, 6- Myoglobin, 7- Molecular weight marker.
(TIF)

S1 Table *B*. *glabrata* miRNA and piRNA pathway proteins retrieved from Vectorbase and their best hit orthologues from NCBI. (DOCX)

S2 Table Primers of the processing machinery of miRNAs and piRNAs in *B*. *glabrata*. (DOCX)

S3 Table Similarity between Bgl-Argonaute and their orthologues of others organisms Protostome and Deuterostome. (DOCX)

S4 Table Similarity between Bgl-Piwi and their orthologues of others organisms Protostome and Deuterostome. (DOCX)

S5 Table Similarity between Bgl-Drosha and their orthologues of others organisms Protostome and Deuterostome. (DOCX)

S6 Table Similarity between Bgl-Dicer and their orthologues of others organisms Protostome and Deuterostome. (DOCX)

S7 Table Sanger sequences of amplicons analysed by CAP3. (DOCX)
