# Supplementary figures and images for: Differential expression of small RNA pathway genes associated with the Biomphalaria glabrata/Schistosoma mansoni interaction
Source: PLoS One. 2017 Jul 18;12(7):e0181483. doi: 10.1371/journal.pone.0181483 (PMC5515444; doi:10.1371/journal.pone.0181483)

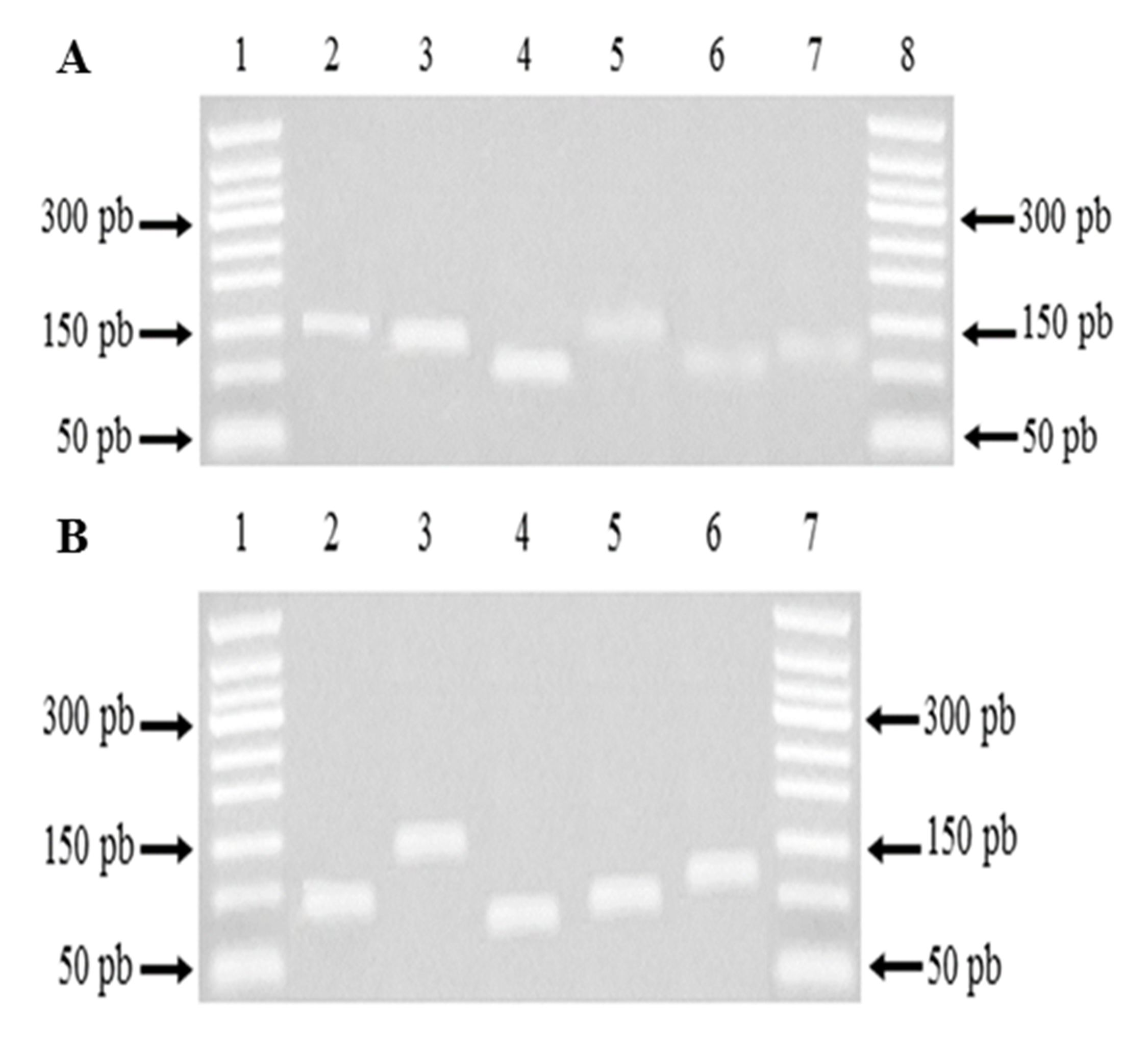

Supplement: S1 Fig — A: 1- Molecular weight marker, 2- Argonaute, 3- Dicer, 4- Drosha, 5- FMR, 6- Loquacious, 7- TDRD, 8- molecular weight marker. B: 1- Molecular weight marker, 2- Piwi, 3- Tudor, 4- SPN, 5- Exportin-5, 6- Myoglobin, 7- Molecular weight marker. (TIF) [file pone.0181483.s001.tif]
