# Supplementary material for: Differential expression of small RNA pathway genes associated with the Biomphalaria glabrata/Schistosoma mansoni interaction
Source: PLoS One. 2017 Jul 18;12(7):e0181483. doi: 10.1371/journal.pone.0181483 (PMC5515444; doi:10.1371/journal.pone.0181483)
Supplement: S1 Table — (DOCX) [file pone.0181483.s002.docx]

**S1 Table. *B. glabrata* miRNA and piRNA pathway proteins retrieved from Vectorbase and their best hit orthologues from NCBI.**

| Gene | Protein ID | length (aa) | Orthologs Blastp NCBI | E-value |
| --- | --- | --- | --- | --- |
| Bgl-Argonaute | BGLB002396-PA | 855 | NP_725341.1 | 0 |
| Bgl-Dicer | BGLB002125-PA | 2165 | NP_524453.1 | 0 |
| Bgl-Drosha | BGLB003167-PA | 1128 | NP_477436.1 | 0 |
| Bgl-Fmr1 | BGLB012977-PA | 376 | NP_731445.1 | 0 |
| Bgl-Loquacious | BGLB009745-PA | 319 | NP_609646.1 | 8e-167 |
| Bgl-TDRD1 | BGLB002484-PA | 3332 | NP_476773.1 | 0 |
| Bgl-PIWI | BGLB010170-PA | 854 | NP_001036627.2 | 0 |
| Bgl-Tudor | BGLB005262-PA | 864 | NP_612021.1 | 0 |
| Bgl-SPN-E | BGLB001557-PA | 1424 | NP_476741.1 | 0 |
| Bgl-Exportin-5 | BGLB008947-PA | 437 | NP_741567.1 | 0.0 |
| Bgl-Myoglobin | BGLB010864-PA | 150 | NP_510079.2 | 4e-97 |
