# Supplementary material for: Differential expression of small RNA pathway genes associated with the Biomphalaria glabrata/Schistosoma mansoni interaction
Source: PLoS One. 2017 Jul 18;12(7):e0181483. doi: 10.1371/journal.pone.0181483 (PMC5515444; doi:10.1371/journal.pone.0181483)
Supplement: S2 Table — (DOCX) [file pone.0181483.s003.docx]

**S2 Table. Primers of the processing machinery of miRNAs and piRNAs in *B. glabrata***

| Gene | Sequences (5’ to 3’) | Product |
| --- | --- | --- |
| Bgl-Argonaute | (F): TTAGCTACCCAGTGCGTCCAG | 148 pb |
|  | (R): TTGGTTCTCGGAATACATGAGG |  |
| Bgl-Dicer | (F): GTCAAGGCAGACTGTGGCTACC | 136 pb |
|  | (R): CTTTGGAACTGAATCCTGCTCG |  |
| Bgl-Drosha | (F): CATTTGCGTTTCCACATTTGTC | 105 pb |
|  | (R): GCGGTAAGAAGTGTGAGTCAGG |  |
| Bgl-Fmr1 | (F): CATGGAAAGCATTAGCAACG | 156 pb |
|  | (R): TCTCTAGGAGGCGGGAAATAAG |  |
| Bgl-Loquacious | (F): CAAGGTCCTCCACATGCCAG | 110 pb |
|  | (R): TTTGCTGCGGCTGTTCGTTTC |  |
| Bgl-TDRD1 | (F): GGTGTCAGGAGAGTGAATCGG | 126 pb |
|  | (R): ACTTGGCAGCACAAATCATCC |  |
| Bgl-PIWI | (F): TGCAACCTTTGATGACTGCTGC | 98 pb |
|  | (R): CCCATCACCAACACCATCTCTG |  |
| Bgl-Tudor-SN | (F): TGCCACTGCCACCACGATAC | 152 pb |
|  | (R): AACACTTCCACCACAAGCCAC |  |
| Bgl-SPN-E | (F): TGGCTAAGTCATCTTTGGTTGC | 92 pb |
|  | (R): AACTGGCAGGTCTCTCAACATG |  |
| Bgl-Exportina 5 | (F): AGACAGGCGGCATTCATTG | 101 pb |
|  | (R): TCCATGAAGTACTCGGGTGAAC |  |
| Bgl-Mioglobina | (F): CAAGCTCGCCAAGTCTCACGTC | 126 pb |
|  | (R): GCTTTGGGTTCGTCACTTCCG |  |
| Bgl-Partner-Drosha | (F): ACCCAGTCCCTTCCAACTTCTC | 123 pb |
|  | (R): CCCACATTGATGGTGAACTCAC |  |
