# Supplementary material for: Differential expression of small RNA pathway genes associated with the Biomphalaria glabrata/Schistosoma mansoni interaction
Source: PLoS One. 2017 Jul 18;12(7):e0181483. doi: 10.1371/journal.pone.0181483 (PMC5515444; doi:10.1371/journal.pone.0181483)
Supplement: S3 Table — (DOCX) [file pone.0181483.s004.docx]

**S3 Table. Similarity between Bgl-Argonaute and their orthologues of others organisms Protostome and Deuterostome.**

| Organism | Protein ID | E-value Blastp | Length (aa) |
| --- | --- | --- | --- |
| *Biomphalaria glabrata* | BGLB002396-PA | N.A | 855 |
| *Aplysia californica* | XP_005107587.1 | 0.0 | 932 |
| *Lottia gigantea* | XP_009064000.1 | 0.0 | 919 |
| *Metaseiulus occidentalis* | XP_003747659.1 | 0.0 | 887 |
| *Ceratitis capitata* | XP_004529840.1 | 0.0 | 982 |
| *Drosophila melanogaster* | NP_725341.1 | 0.0 | 984 |
| *Apis dorsata* | XP_006622112.1 | 0.0 | 818 |
| *Homo sapiens* | NP_036286.2 | 0.0 | 859 |
| *Mus musculus* | NP_700451.2 | 0.0 | 860 |
| *Crassostrea gigas* | EKC19600.1 | 0.0 | 1002 |
| *Caenorhabditis elegans* | NP_001257239.1 | 0.0 | 1023 |
| *Rattus norvegicus* | NP_067608.1 | 0.0 | 863 |
| *Danio rerio* | NP_001289151.1 | 0.0 | 873 |
| *Canis lupus familiaris* | XP_005629013.1 | 0.0 | 860 |
| *Musca domestica* | XP_005175308.1 | 0.0 | 987 |
| *Bombus terrestris* | XP_003400152.1 | 0.0 | 928 |
| *Gallus gallus* | XP_004940123.1 | 0.0 | 863 |
| *Bos taurus* | NP_991363.1 | 0.0 | 860 |
| *Schistosoma japonicum* | Sjp_0044720.1 | 0.0 | 987 |
| *Schistosoma mansoni* | Smp_198380.1 | 0.0 | 928 |
