# Supplementary material for: Differential expression of small RNA pathway genes associated with the Biomphalaria glabrata/Schistosoma mansoni interaction
Source: PLoS One. 2017 Jul 18;12(7):e0181483. doi: 10.1371/journal.pone.0181483 (PMC5515444; doi:10.1371/journal.pone.0181483)
Supplement: S4 Table — (DOCX) [file pone.0181483.s005.docx]

**S4 Table. Similarity between Bgl-Piwi and their orthologues of others organisms Protostome and Deuterostome.**

| Organism | Protein ID | E-value Blastp | Length (aa) |
| --- | --- | --- | --- |
| *Biomphalaria glabrata* | BGLB010170-PA | N.A | 854 |
| *Aplysia californica* | XP_005096149.1 | 0.0 | 918 |
| *Lottia gigantea* | XP_009064630.1 | 0.0 | 791 |
| *Nematostella vectensis* | XP_001641994.1 | 0.0 | 871 |
| *Hydra vulgaris* | NP_001274302.1 | 0.0 | 889 |
| *Drosophila melanogaster* | NP_476875.1 | 2e-170 | 843 |
| *Homo sapiens* | NP_004755.2 | 0.0 | 861 |
| *Mus musculus* | NP_067286.1 | 0.0 | 862 |
| *Crassostrea gigas* | EKC35279.1 | 0.0 | 885 |
| *Danio rerio* | NP_899181.1 | 0.0 | 858 |
| *Canis lupus familiaris* | XP_534638.2 | 0.0 | 861 |
| *Rattus norvegicus* | XP_008764202.1 | 0.0 | 848 |
| *Gallus gallus* | NP_001092322.1 | 0.0 | 867 |
| *Bos taurus* | XP_618020.4 | 0.0 | 861 |
| *Aedes aegypti* | XP_001652945.1 | 0.0 | 944 |
| *Bombus terrestris* | XP_003400353.1 | 0.0 | 902 |
| *Musca domestica* | XP_005183556.1 | 0.0 | 929 |
