# Supplementary material for: Differential expression of small RNA pathway genes associated with the Biomphalaria glabrata/Schistosoma mansoni interaction
Source: PLoS One. 2017 Jul 18;12(7):e0181483. doi: 10.1371/journal.pone.0181483 (PMC5515444; doi:10.1371/journal.pone.0181483)
Supplement: S5 Table — (DOCX) [file pone.0181483.s006.docx]

**S5 Table. Similarity between Bgl-Drosha and their orthologues of others organisms Protostome and Deuterostome.**

| Organism | Protein ID | E-value Blastp | Length (aa) |
| --- | --- | --- | --- |
| *Biomphalaria glabrata* | BGLB003167-PA | N.A | 1128 |
| *Tribolium castaneum* | XP_008199088.1 | 0.0 | 1176 |
| *Apis dorsata* | XP_006618766.1 | 0.0 | 1307 |
| *Apis mellifera* | XP_006558454.1 | 0.0 | 1307 |
| *Bombus terrestris* | XP_003394274.1 | 0.0 | 1306 |
| *Drosophila melanogaster* | NP_477436.1 | 0.0 | 1327 |
| *Homo sapiens* | XP_005248351.1 | 0.0 | 1306 |
| *Mus musculus* | XP_006520084.1 | 0.0 | 1393 |
| *Crassostrea gigas* | EKC20603.1 | 0.0 | 1354 |
| *Caenorhabditis elegans* | NP_001122460.2 | 2e-87 | 1081 |
| *Danio rerio* | NP_001103942.1 | 0.0 | 1289 |
| *Rattus norvegicus* | NP_001101125.2 | 0.0 | 1373 |
| *Canis lupus familiaris* | XP_854135.2 | 0.0 | 1373 |
| *Bos taurus* | XP_591998.4 | 0.0 | 1330 |
| *Musca domestica* | XP_005186977.1 | 0.0 | 1384 |
| *Gallus gallus* | NP_001006379.1 | 0.0 | 1336 |
| *Schistosoma mansoni* | Smp_142510.2 | 1.3e-124 | 1577 |
| *Schistosoma japonicum* | Sjp_0048900.1 | 3.7e-135 | 1611 |
