# Supplementary material for: Differential expression of small RNA pathway genes associated with the Biomphalaria glabrata/Schistosoma mansoni interaction
Source: PLoS One. 2017 Jul 18;12(7):e0181483. doi: 10.1371/journal.pone.0181483 (PMC5515444; doi:10.1371/journal.pone.0181483)
Supplement: S6 Table — (DOCX) [file pone.0181483.s007.docx]

**S6 Table. Similarity between Bgl-Dicer and their orthologues of others organisms Protostome and Deuterostome.**

| Organism | Protein ID | E-value Blastp | Length (aa) |
| --- | --- | --- | --- |
| *Biomphalaria glabrata* | BGLB002125-PA | N.A | 2165 |
| *Aplysia californica* | XP_005106232.1 | 0.0 | 2495 |
| *Drosophila melanogaster* | NP_524453.1 | 0.0 | 2249 |
| *Mus musculus* | NP_683750.2 | 0.0 | 1906 |
| *Apis dorsata* | XP_006618601.1 | 3e-172 | 1954 |
| *Ceratitis capitata* | XP_004523314.1 | 7e-142 | 2306 |
| *Metaseiulus occidentalis* | XP_003745061.1 | 9e-141 | 1863 |
| *Caenorhabditis elegans* | NP_498761.2 | 6e-111 | 1910 |
| *Danio rerio* | NP_001154925.1 | 0.0 | 1865 |
| *Canis lupus familiaris* | XP_868526.3 | 0.0 | 1924 |
| *Rattus norvegicus* | XP_008774532.1 | 0.0 | 1918 |
| *Bos taurus* | NP_976235.1 | 0.0 | 1923 |
| *Musca domestica* | XP_005179924.1 | 4e-149 | 2385 |
| *Schistosoma mansoni* | Smp_169750.2 | 2.5e-161 | 2541 |
