# Supplementary material for: Differential expression of small RNA pathway genes associated with the Biomphalaria glabrata/Schistosoma mansoni interaction
Source: PLoS One. 2017 Jul 18;12(7):e0181483. doi: 10.1371/journal.pone.0181483 (PMC5515444; doi:10.1371/journal.pone.0181483)
Supplement: S7 Table — (DOCX) [file pone.0181483.s008.docx]

**S7 Table. Sanger Sequences of amplicons analysed by CAP3.**

| Gene | Strand | Sequence of Contigs (5’ to 3’) |
| --- | --- | --- |
| Bgl-Argonaute | - | CCCGGCCTAGGGCATAGTATACTGTTGACTCCCCAAGCTTAACATTGATCTTAAGGCAGAGATTGGAAAGAGTCTGTGGAGTGGTTTTGTTCACATTCTTGGCCTGGACGCACTGGGTAGCTAAAACGG |
| Bgl-Dicer | + | GGACACGGGACAATAATGGAATCGCATAATGAATTAGAATAGAATGGGACTTTGTGGACAAAGTAATTGATTTTATAAAAATAGAGAAACGAGCAGGATTCAGTTCCAAAGAAGGATTTTAAAAGGT |
| Bgl-Drosha | - | GGCAATAAAGGGATTTCTATCACAACTTGTAGTCTATGGAATTCTCTAGTACATCCAGACAAATGTGGAAACGCAAAGCTAAAAAGCC |
| Bgl-Fmr1 | - | CGGGCAAATAAGGTTCACTTGGATCATCTCAAGGAAGTGGAACAGTTGCGCCAAGCTAAACTTGAAATTGACCAACAGCTTAAGTCATTATCAGGGCCCCAGCCTGGCTCTTATTTCCCGCCTCCTAGAGAAGACCTGTGATATGAAATCTGGATGGGCGTCTTTCTGTTTTTTTTGTCAGCACTTCTTCTCTCCCATGAATCAGCCTATACTT |
| Bgl-Loquacious | + | GGGGGGTTTTTCCATCAGTTACCTTTTTGTCACAAACTTTCCCAATTTGATGTTGCAGATGAATTCTCTGGCATGTGGAGGACCTGAGCCCGGTCTGGCGTCTAGATGATTAAAGGGAGGGCCCA |
| Bgl-TDRD1 | - | GGGCCAGTCTATCTATCAGCAGTATTATCCAGACTTCATATGACTCAGGACATGAGGCCATTACACAACTGTGTCCCAGGGATGATTTGTGCTGCCAAGTAATATCGGAATTTAAAAAGAAGGCTATCTCGTGACTAGGAGGGGGGAAATTCCTCATTTATTGCCCTCTTCAATCTTTTCTCAAACGATAACGTGGA |
| Bgl-PIWI | - | AATTCCTTTTTTGCGGGCAGTGCCTTGTTCACATCATGATATTTCCTAAGTGCAGCAGTCATCAAAGGTTGCAATTGGAAAAAAGATGATGTGAACAAGGCACTGCCGCAAAAGATAATTCTTAAACAGAGGGGGTGTGGGGGGGGGGA |
| Bgl-Tudor-SN | + | TTTTGGGCCTGTGTATCAGTCCAAGTGATGAGATGCTAAGCAAGATGCTGTTGATGCTCTTTACAATGACATCCTGAACAAGACAATGCTGCTCAATATTGAGTATCGTGGTGGCAGTGGCAAGAGCATCAACAGCATCTTGCTTAGCATCTTCATCACTTGGGACTGATACACAGGCAAGTGTATATTCAGTGGCTTGTGGTGGAAGTGTACAGCAGATGCTGTTGATGCTCTTTACAATGACATCCTGAACAAGACAATGCTGCTCAATATTGAGTATCGTGGTGGCAGTGGCAA |
| Bgl-SPN-E | + | GGGGGATTCTCTGGTGGCTTGGCACACAGAGTTGATAGAAGTTGGCATGTTGAGAGACCTGCCAGTAGATCGGGGGAGTTGGATAGTCAACAAAGAAAACCTTAGCAACCAAAGATGACTTAGCA |
| Exportin 5 | - | GGGGAATTATTAATCTTCTGATGACCACAACTCCTTGACCGGTGCAATAACTTCCTCAATGAATGCCGCCTGTCACCATGAGGGGCTCGAAGATTCTATAAGGCTTTGGTTCCTCAAAAAAAAAAAGGAAATTTTTTCCAACAAATTACTCAT |
| Myoglobin | + | AAAAACGGACTCGAGTTCTTCGGACTCTGCACAGAACATTCACAGCTTCATTGAAAGTGCTCTTGGAGTCGGTGCCGGAAGTGACGAACCCAAAGCACTTCATGGGGTGGGACAGGACAGGTGGAGGGGGAAGGAGGATTGGGTCCGGTTTTCGGGTGGAAGGGAGGGGTGGGTTGGCTTCGTCTTTCCCAAAAAAAAACCTCCCCGCATAACACAAAAAAAAAAACCACGACAACTAAACTCACTACTA |
